# Impact of COVID-19 pandemic on mental health: An international study

**DOI:** 10.1371/journal.pone.0244809

**Published:** 2020-12-31

**Authors:** Andrew T. Gloster, Demetris Lamnisos, Jelena Lubenko, Giovambattista Presti, Valeria Squatrito, Marios Constantinou, Christiana Nicolaou, Savvas Papacostas, Gökçen Aydın, Yuen Yu Chong, Wai Tong Chien, Ho Yu Cheng, Francisco J. Ruiz, Maria B. Garcia-Martin, Diana P. Obando-Posada, Miguel A. Segura-Vargas, Vasilis S. Vasiliou, Louise McHugh, Stefan Höfer, Adriana Baban, David Dias Neto, Ana Nunes da Silva, Jean-Louis Monestès, Javier Alvarez-Galvez, Marisa Paez-Blarrina, Francisco Montesinos, Sonsoles Valdivia-Salas, Dorottya Ori, Bartosz Kleszcz, Raimo Lappalainen, Iva Ivanović, David Gosar, Frederick Dionne, Rhonda M. Merwin, Angelos P. Kassianos, Maria Karekla

**Affiliations:** 1 Division of Clinical Psychology & Intervention Science, Department of Psychology, University of Basel, Basel, Switzerland; 2 Department of Health Sciences, European University Cyprus, Nicosia, Cyprus; 3 Psychological Laboratory, Faculty of Public Health and Social Welfare, Riga Stradiņš University, Riga, Latvia; 4 Kore University Behavioral Lab (KUBeLab), Faculty of Human and Social Sciences, Kore University of Enna, Enna, Italy; 5 Department of Social Sciences, School of Humanities and Social Sciences, University of Nicosia, Nicosia, Cyprus; 6 Department of Nursing, Cyprus University of Technology, Limassol, Cyprus; 7 Cyprus Institute of Neurology and Genetics, Nicosia, Cyprus; 8 Department of Psychological Counseling and Guidance, Faculty of Education, Hasan Kalyoncu University, Gaziantep, Turkey; 9 The Nethersole School of Nursing, Faculty of Medicine, The Chinese University of Hong Kong, Hong Kong; 10 Department of Psychology, Fundación Universitaria Konrad Lorenz, Bogotà, Columbia; 11 Faculty of Psychology, University of La Sabana, Chía, Columbia; 12 School of Applied Psychology, University College Cork, Cork, Ireland; 13 School of Psychology, University College Dublin, Dublin, Ireland; 14 Medical University Innsbruck, Innsbruck, Austria; 15 Department of Psychology, Babeş-Bolyai University (UBB), Cluj-Napoca, Romania; 16 Instituto Superior de Psicologia Aplicada (ISPA), Instituto Universitário; APPsyCI—Applied Psychology Research Center Capabilities & Inclusion, Lisboa, Portugal; 17 Faculdade de Psicologia, Alameda da Universidade, Universidade de Lisboa, Lisboa, Portugal; 18 LIP/PC2S, Université Grenoble Alpes, Grenoble, France; 19 Department of Biomedicine, Biotechnology and Public Health, University of Cadiz, Cadiz, Spain; 20 Instituto ACT, Madrid, Spain; 21 Department of Psychology, European University of Madrid, Madrid, Spain; 22 Department of Psychology and Sociology, University of Zaragoza, Zaragoza, Spain; 23 Vadaskert Child and Adolescent Psychiatric Hospital, Budapest, Hungary; 24 Private Pratice, Poland; 25 Department of Psychology, University of Jyväskylä, Jyväskylä, Finland; 26 Clinic for Psychiatry, Clinical Center of Montenegro, Podgorica, Montenegro; 27 Ljubljana University Medical Centre, Ljubljana, Slovania; 28 Département de Psychologie, Université du Québec à Trois-Rivières, Trois-Rivières, Canada; 29 Department of Psychiatry and Behavioral Science, Duke University, Durham, North Carolina, United States of America; 30 Department of Psychology, University of Cyprus, Nicosia, Cyprus; University of the Witwatersrand, SOUTH AFRICA

## Abstract

**Background:**

The COVID-19 pandemic triggered vast governmental lockdowns. The impact of these lockdowns on mental health is inadequately understood. On the one hand such drastic changes in daily routines could be detrimental to mental health. On the other hand, it might not be experienced negatively, especially because the entire population was affected.

**Methods:**

The aim of this study was to determine mental health outcomes during pandemic induced lockdowns and to examine known predictors of mental health outcomes. We therefore surveyed n = 9,565 people from 78 countries and 18 languages. Outcomes assessed were stress, depression, affect, and wellbeing. Predictors included country, sociodemographic factors, lockdown characteristics, social factors, and psychological factors.

**Results:**

Results indicated that on average about 10% of the sample was languishing from low levels of mental health and about 50% had only moderate mental health. Importantly, three consistent predictors of mental health emerged: social support, education level, and psychologically flexible (vs. rigid) responding. Poorer outcomes were most strongly predicted by a worsening of finances and not having access to basic supplies.

**Conclusions:**

These results suggest that on whole, respondents were moderately mentally healthy at the time of a population-wide lockdown. The highest level of mental health difficulties were found in approximately 10% of the population. Findings suggest that public health initiatives should target people without social support and those whose finances worsen as a result of the lockdown. Interventions that promote psychological flexibility may mitigate the impact of the pandemic.

## Introduction

The COVID-19 global pandemic caused by the severe acute respiratory syndrome coronavirus 2 (SARS-COV-2) virus triggered governmentally mandated lockdowns, social distancing, quarantines and other measures in the interest of public health. The mandated lockdowns abruptly and dramatically altered people’s daily routines, work, travel, and leisure activities to a degree unexperienced by most people living outside of war zones. Simultaneously, the highly contagious, yet invisible virus transformed previously neutral situations to perceived potentially dangerous ones: social interaction, touching one’s face, going to a concert, shaking someone’s hand, and even hugging grandparents. Given these changes and looming threat, increases in anxiety and depression can be expected [1]. Indeed, common psychological reactions to previous quarantines include post-traumatic symptoms, confusion, and anger [2], though these data stem from quarantines of specific regions or a subgroup of exposed people, such as medical professionals. It therefore remains an empirical question whether such patterns are consistent when entire populations across the globe are simultaneously affected.

For most people, it stands to reason that governmentally mandated lockdowns decrease their activity levels and the number of stimuli experienced compared to pre-lockdown levels. The impact of reducing activities, stimuli and routines on the population is unknown, but various analogue situations can be used to make predictions, like death of a spouse [3]; hearing loss [4]; job loss [5]; long duration expeditions [6]; poor acculturation [7]; and even ageing when combined with loneliness [8]. Each of these situations is associated with increases in psychological distress. This reduction of stimulations may lead to boredom and reductions in reinforcement, which has been associated with depression [9]. The sum total of these literatures, and some evidence from country specific studies on COVID-19 suggests that for some people, the mental distress in the form of stress, depression, and negative affect are likely reactions to the lockdown; therefore, people’s wellbeing is likely to suffer. Indeed, increased loneliness, social isolation, and living alone are associated with increased mortality [10]–the exact effect that mandated lockdown and social distancing rules aimed to counteract.

Alternately, the planned slowing down of daily routines can be beneficial. For example, vacations and weekends are highly sought-after–if not always achieved–periods of relaxation and stress reduction [11]. Likewise, some religious and spiritual traditions encourage simplicity, mindfulness, and solitude with the goal of increasing wellbeing [12]. It is therefore conceivable that for some people the lockdown could offer a reprieve from daily hassles and stress and even lead to increases in wellbeing. It is therefore equally important to identify protective factors that can buffer against the negative effects of the lockdown.

Although nearly all people around the globe have been subject to some form of lockdown measures to contain the COVID-19 response, variations exist with respect to how each person is confined, even within a single country. For instance, during the COVID-19 pandemic some people were allowed to go to work, whereas others were required to work exclusively from home. For various reasons, some people had difficulty obtaining some basic supplies. Further, some were thrust into the situation of taking care of others (e.g., children, due to closing of schools). Finally, some people lost income as a result of the lockdown, and this is a known risk-factor for poor mental health [13, 14]. Finally, a lockdown may be experienced differently the longer it continues and potentially when in confined spaces [2]. All of these lockdown-specific features may have an impact on one’s mental health, but to date it remains inadequately explored.

As the risk of the pandemic continues, it is important to understand to what degree the virus-induced uncertainty and the lockdown-induced changes in daily routines impact stress, depression, affect, and wellbeing. Towards this end, it is important to identify factors that can mitigate potential negative psychological effects of pandemics and lockdowns. Various social and psychological factors have been identified in other contexts that may also help build resilience in large-scale pandemics such as COVID-19. On the social level, one such candidate is social support, which has repeatedly been found to positively impact mental health and wellbeing [15–18]. Another social factor is the family climate and family functioning, which clearly impacts people’s mental health [19, 20]. Psychological factors such as mindfulness and psychologically flexible response styles (as opposed to rigid and avoidant response styles) are behavioral repertoires that have previously been shown to buffer the impact of stress and facilitate wellbeing [21–24].

Given the scope of the COVID-19 pandemic, it is crucial to better understand how a pandemic and associated lockdowns impact on mental health. Thus, the aim of this study was to determine mental health outcomes and to examine known predictors of outcomes to identify psychological processes and contextual factors that can be used in developing public health interventions. It can be assumed, but remains untested, that those with risks in social-demographic factors, living conditions, social factors and psychological factors have more severe reactions to the lockdown. We therefore tested whether outcomes of stress, depression, affect, and wellbeing were predicted by country of residence, social demographic characteristics, COVID-19 lockdown related predictors, social predictors, and psychological predictors.

## Methods

### Participants

The inclusion criteria were ≥18 years of age and ability to read one of the 18 languages (English, Greek, German, French, Spanish, Turkish, Dutch, Latvian, Italian, Portuguese, Finnish, Slovenian, Polish, Romanian, Hong Kong, Hungarian, Montenegrin, & Persian.). There were no exclusion criteria. People from all countries were eligible to participate.

### Procedure

Ethics approval was obtained from the Cyprus National Bioethics Committee (ref.: EEBK EΠ 2020.01.60) followed by site approvals from different research teams involved in data collection. All participants provided written informed consent prior to completing the survey (computer-based, e.g., by clicking “yes”).

A population based cross-sectional study was conducted in order to explore how people across the world reacted to the COVID-19. The anonymous online survey was distributed using a range of methods. Universities emailed the online survey to students and academic staff and also posted the survey link to their websites. In addition, and in order to broaden the sample to older age groups and to those with different socio-demographic characteristics, the survey was disseminated in local press (e.g., newspapers, newsletters, radio stations), in social media (e.g., Facebook, Twitter, etc.), in professional networks, local hospitals and health centers and professional groups’ email lists (e.g., medical doctors, teachers, engineers, psychologists, government workers), and to social institutions in the countries (e.g., churches, schools, cities/townships, clubs, etc.).

Data were collected for two months between 07th April and 07th June 2020. The majority of countries where data were collected had declared a state of emergency for COVID-19 during this time.

### Measures

Well validated and established measures were used to assess constructs. When measures did not already exist in a language, they were subject to forward and backward translation procedures. Well-validated measures of predictors and outcomes and items measuring COVID-19 related characteristics were selected after a consensus agreement among the members of this study.

### Predictors

#### Country

Respondents’ countries were coded and entered as predictors.

#### Socio-demographic status

Participants responded to questions related to their socio-demographic characteristics including their age, gender, country of residence, marital status, employment status, educational level, whether they have children as well as their living situation.

#### Lockdown variables

Participants responded to questions related to lockdown including length of lockdown, whether they need to leave home for work, any change in their finances, whether they were able to obtain basic supplies, the amount of their living space confined in during the lockdown. They were also asked whether they, their partner, or a significant other was diagnosed with COVID-19.

#### Social factors

Social factors were measured using the Brief Assessment of Family Functioning Scale (BAFFS; [25]) and the Oslo Social Support Scale (OSSS; [26]). The BAFFS items are summed to produce a single score with higher scores indicating worse family functioning. The OSSS items are summed up and provide three levels types of social support: low (scored 3–8), moderate (scored 9–11) and high (scored 12–14).

#### Psychological factors

Psychological factors including mindfulness and psychological flexibility. Mindfulness was measured using the Cognitive Affective Mindfulness Scale (CAMS; [27]). The CAMS produces a single score with higher scores indicating better mindfulness qualities. Psychological flexibility (e.g., hold one’s thoughts lightly, be accepting of one’s experiences, engage in what is important to them despite challenging situations) was measured using the Psyflex scale [28]. The Psyflex produces a single score with higher scores indicating better psychological flexibility qualities.

### Outcomes

#### Stress

Stress was measured using the Perceived Stress Scale (PSS; [29]). The PSS assesses an individual’s appraisal of how stressful situations in their life are. Items ask about people’s feelings and thoughts during the last month. A total score is produced, with higher scores indicating greater overall distress.

#### Depression

Depressive symptomatology was assessed using two items from the disengagement subscale of the Multidimensional State Boredom Scale (MSBS; [30]). These items assessed wanting to do pleasurable things but not finding anything appealing (i.e., boredom), as well as wasting time. Based on concepts of reinforcement deprivation (i.e., lack of access to or engagement with positive stimuli) that is known to contribute to depression, we added an item that measured how rewarding or pleasurable people found the activities that they were engaging in (i.e., reinforcement). Higher scores indicated higher depressive symptomatology.

#### Positive affect/ negative affect

The Positive And Negative Affect Scale (PANAS) was used to measure affect [31]. The original version of the questionnaire was used with five additional items: bored, confused, angry, frustrated and lonely. All items were scored on a 5-point Likert type scale, ranging from 1 = very little/not at all to 5 = extremely and summed up so that higher scores in the positive-related items indicating higher positive affect and higher scores in the negative-related items indicating higher negative affect. In order to capture additional dimensions of negative affect believed to be relevant to the COVID-19 lockdowns, we additionally added five items: bored, confused, angry, frustrated, lonely.

#### Wellbeing

Wellbeing was assessed using the Mental Health Continuum Short Form (MHC-SF; [32]); which assesses three aspects of wellbeing: emotional, psychological, and social. The MHC-SF produces a total score and scores for each of the three aspects of wellbeing. The MHC-SF can also be scored to produce categories of languishing (i.e., low levels of emotional, psychological, and social well-being), flourishing (i.e., high levels of emotional psychological and social well-being almost every day), and moderately mentally healthy (in between languishing and flourishing).

### Statistical analysis

The mean and standard deviation was calculated for dependent variables that follow the normal distribution while the median and interquartile range (IQR) were computed for non-normally distributed data. Bivariable association between an outcome variable and each predictor was investigated with ANOVA test for categorical predictor and univariable linear regression for numerical predictor. Linear mixed-effect model with random effect for country was performed to consider simultaneously several predictors in the same model and to account for the variation in outcome variable between countries. Four separate linear mixed-effect models were used for each outcome variable, one for each set of socio-demographic, lockdown, social and psychosocial predictors and multicollinearity for each set of predictors was investigated with the variation inflation criterion (VIF). Standardized regression coefficients were computed as effect size indices to measure the strength of the association between predictor variables and outcome variables. The comparison between the country mean and overall mean for each outcome variable was estimated though a linear regression model with dependent variable the mean centering outcome and predictor the country. Cohen’s d effect size of the standardize difference between country mean and the overall mean was computed as a measure of the magnitude of the difference between the two means.

The whole sample was used in linear mixed-effect models while for the comparison of country mean to the overall mean was used the sample from countries with sample size ≥100. The R packages *lme4* and *effect sizes* were used for fitting the linear mixed effect model and to compute the standardized regression coefficients of the linear mixed effect models [33]. Significance test and confidence intervals were calculated at a significance level of 0.05. The following cut-off values were used for the evaluation of the effect sizes: ‘tiny’ ≤0.05, ‘very small’ from 0.05 to ≤0.10, ‘small’ from 0.10 to ≤ 0.20, ‘medium’ from 0.20 to ≤ 0.30, ‘large’ from 0.30 to ≤ 0.40 and ‘very large’ > 0.40 [34].

## Results

### Descriptive

Participants were n = 9,565 people from 78 countries. See supporting information for a participation flowchart (S1 Appendix). The countries with the largest samples were: Latvia (n = 1285), Italy (n = 962), Cyprus (n = 957), Turkey (n = 702), Switzerland (n = 550), Hong Kong (n = 516), Colombia (n = 485), Ireland (n = 414), Austria (n = 368), Romania (n = 339), Portugal (n = 334), France (n = 313), Spain (n = 296), Germany (n = 279), Hungary (n = 273), Greece (n = 270), USA (n = 268), Finland (n = 157), Montenegro (n = 147), Poland (n = 135), United Kingdom (n = 100), Slovenia (n = 77), and Canada (n = 60). The remaining countries are listed in the supporting information (S1 Table).

### Outcome variables

The means, standard deviations, and where appropriate percentage of participants within categories of the five outcome variables can be seen in Table 1.

**Table 1:** Descriptive statistics for outcomes and predictors (continuous variables).

	Mean (M) (SD)	%
Outcomes		
Stress	17.1 (7.5)	
Low		33.0%
Moderate		55.9%
High		11.1%
Depression Symptoms	6.6 (2.3)	
Lack of Reinforcement	2.1 (1.0)	25.8%^a^
Boredom	2.4 (1.1)	32.4%^a^
Waste Time	2.4 (1.1)	47.4%^a^
Positive Affect	30.0 (8.1)	
Negative Affect	19.6 (7.8)	
Wellbeing	41.0 (14.0)	
Languishing		10.1%
Moderately Mentally Healthy		50.0%
Flourishing		39.9%
Predictors		
Family Functioning	5.5 (1.9)	
Social Support	9.9 (2.1)	
Mindfulness	26.7 (3.2)	
Psychological Flexibility	21.8 (4.1)	

*Note*: Possible ranges of the scales were as follows: Stress (PSS; 0–40); Reinforcement (1–4); Boredom (MSBS; 1–4); Positive Affect (PANAS-P; 10–50); Negative Affect (PANAS-N, plus; 15–75); Wellbeing (MHC-SF; 0–70); Family Functioning (BAFFS; 3–12); Social Support (OSSS; 3–14); Mindfulness (CAMS-R; 0–40); Psychological Flexibility (Psyflex; 6–30)

^a^ = % of participants who responded with “lot” or “extremely”.

### Predictor variables

#### Countries

A full list of countries can be found in the supporting information (S1 Table).

#### Socio-demographic status

The mean age was 36.9 (13.3) years. A majority of participants were female (77.7%), approximately a fifth male (22.0%), and small minority identified as other (0.3%). More than half of the respondents were either in a relationship (25.7%) or married (36.1%), almost a third were single (30.8%), and the rest were either divorced (5%), widower (1.1%) or other (1.3%). Participants indicated that they lived: alone (14.6%), with both parents (20.8%), one parent (5.1%), with their own family including partner and children (54.1%), or with friends or roommates (5.5%). Less than half of respondents had children (40.8%). Approximately half of the participants were working full time (53.4%), almost a fifth were working part-time (17.5%), 23.2% were unemployed and a small minority were either on parental leave (2.2%) or retired (3.7%).

#### COVID-19 lockdown variables

At the time of responding, participants were in lockdown or self-isolation for a median of 5.0 (3.0 IQR) weeks. Most people indicated that they had not been infected with COVID-19 (88.0%), a small minority indicated they had been infected (1.4%) and the rest had symptoms but were unsure (10.6%). Similar patterns were seen with reported infection rates of partners (no: 92.2%, yes: 0.7%, unsure: 7.1%) and of people close to them (no: 86.0%; yes: 5.6%; unsure: 8.4%). With respect to leaving the house for work, almost half (47.7%) indicated that this never occurred, 7.7% indicated leaving only once, whereas an almost equal number indicated leaving a couple times per week (23.7%) or more than three times per week (21.0%). Nearly all participants indicated they were able to obtain all the basic supplies they needed (93.5%). Participants reported having a median inner living space of 90.0 square meters (80.0 IQR) and median outdoor space of 20.0 square meters (192.1 IQR). Finally, with respect to finances, more than half indicated that their financial situation remained about the same (57.9%), a minority indicated it improved (8.9%), and a third reported that their finances had gotten worse (33.3%).

#### Social and psychological predictors

Mean values of the other predictors (i.e., social predictors and psychological predictors) can be seen in Table 1.

### Multivariate analyses

#### Stress

Results of multivariate analyses for the outcome of stress can be seen in Table 2. The largest protective factor against stress was social support (high support vs low support (-3.35, 95%CI, -3.39 to -2.92), with a very large effect size). Positive predictors of stress with large effect sizes were being female (2.42, 95%CI, 2.07 to 2.77) and worsening of finances (2.32, 95%CI, 1.68 to 2.96), whereas psychological flexibility buffered this response (-0.65, 95%CI, -0.69 to -0.62). Higher education levels were also associated with lower levels of stress, with a large effect size (see Table 2). Moderate effect sizes for predictors associated with less stress were older age (-0.13, 95%CI, -0.14, -0.11) and mindfulness (-0.69, 95%CI, -0.74, -0.64). Moderate effect sizes of predictors associated with more stress were worse family functioning (0.98, 95%CI, 0.90, 1.06) and not being able to obtain all basic supplies (1.82 95%CI, 1.12, 2.52).

**Table 2:** Predictors of stress.

	PSS Score	
	Coefficient (95% CI)	Effect Size (95% CI)
**Socio Demographic Predictors**		
Sex		
Male	Ref	Ref
Female	2.42 (2.07, 2.77)	0.34 (0.29, 0.39)
Age	-0.13 (-0.14, -0.11)	-0.23 (-0.26, -0.19)
Employment		
Working (full time)	Ref	Ref
Working (part time)	0.23 (-0.18, 0.64)	0.03 (-0.02, 0.09)
Unemployed	1.08 (0.66, 1.51)	0.15 (0.09, 0.21)
On parental leave	-0.05 (-1.03, 0.94)	-0.01 (-0.14, 0.13)
Retired	-0.39 (-1.25, 0.58)	-0.05 (-0.17, 0.07)
Education		
Primary School	Ref	Ref
High School	-2.55 (-4.18, -0.92)	-0.36 (-0.60, -0.13)
Some College/University	-2.11 (-3.74, -0.47)	-0.30 (-0.53, -0.07)
Graduated from College/University	-2.49 (-4.10, -0.87)	-0.36 (-0.59, -0.13)
Master/Postgraduate	-2.81 (-4.42, -1.19)	-0.40 (-0.63, -0.17)
Doctoral level	-2.50 (-4.17, -0.82)	-0.36 (-0.60, 0.17)
Other	-1.78 (-3.60, 0.03)	-0.26 (-0.51, 0.00)
Marital status		
Single	Ref	Ref
In a relationship/engaged	0.20 (-0.24, 0.64)	0.03 (-0.03, 0.09)
Married	0.11 (-0.46, 0.69)	0.02 (-0.06, 0.10)
Divorced	-0.74 (-1.53, 0.03)	-0.10 (-0.21, 0.00)
Widower	0.50 (-0.95, 1.95)	0.07 (-0.13, 0.27)
Other	0.30 (-0.99, 1.59)	0.04 (-0.14, 0.22)
Living situation		
Live alone	Ref	Ref
Live with parents	-0.18 (-0.35, 0.73)	-0.03 (-0.10, 0.05)
Live with one of parents	0.96 (0.29, 1.80)	0.13 (0.03, 0.24)
Live with my own family	-0.70 (-1.37, -0.26)	-0.10 (-0.17, -0.02)
Live with friends/roommates	-0.05 (-0.66, 0.81)	-0.01 (-0.11, 0.09)
Having children		
Yes	Ref	Ref
No	-0.72 (-1.18, -0.26)	-0.10 (-0.16, -0.04)
**Lockdown Predictors**		
Weeks in quarantine/self-isolation	0.05 (-0.00, 0.14)	0.00 (0.00, 0.05)
Leave for work		
No	Ref	Ref
Once only	0.14 (-0.51, 0.79)	0.02 (-0.07, 0.11)
A couple of times	-0.97 (-1.41, -0.53)	-0.13 (-0.19, -0.07)
More than 3 times per week	-1.03 (-1.50, -0.56)	-0.14 (-0.21, -0.08)
Changes in finance		
Have gotten better	Ref	Ref
Stayed about the same	0.54 (-0.06, 1.15)	0.07 (-0.01, 0.15)
Have gotten worse	2.32 (1.68, 2.96)	0.31 (0.23, 0.40)
Obtain all basic supplies		
Yes	Ref	Ref
Νο	1.82 (1.12, 2.52	0.24 (0.15, 0.34)
Inner living space (m2)	-0.02 (-0.03, -0.00)	-0.03 (-0.06, -0.01)
Outer space (m2)	-0.00 (-0.00, 0.00)	-0.01 (-0.03, 0.01)
**Social Predictors**		
Family Functioning Score	0.98 (0.90, 1.06)	0.24 (0.22, 0.26)
Perceived Social support		
Low	Ref	Ref
Moderate	-2.09 (-2.45, -1.72)	-0.27 (-0.32, -0.23)
High	-3.35 (-3.79, -2.92)	-0.44 (-0.49, -0.38)
**Psychological Predictors**		
Mindfulness	-0.69 (-0.74, -0.64)	-0.29 (-0.31, -0.27)
Psychological Flexibility	-0.65 (-0.69, -0.62)	-0.36 (-0.38, -0.34)

*Note*: Effect size interpretation: tiny (0–0.05); very small (0.05–0.10); small (0.10–0.20); medium (0.20–0.30); large (0.30–0.40); very large (>.40); Color code: light green–dark green (tiny–very large positive effect); light red–dark red (tiny–very large negative effect)

Differences in reported levels of stress across countries were largely negligible, with the exception of two countries that reported higher levels of stress (Hong Kong (2.85, 95%CI, 2.22, 3.49) and Turkey (2.47, 95%CI, 1.93, 3.02)) and two that reported lower levels of stress (Portugal (-2.50, 95%CI, -3.29, -1.71) and Montenegro (-3.30, 95%CI, -4.49, -2.11)) than the average stress level across all countries. See supporting information for information on each country (S2–S6 Tables).

#### Depression

Results of multivariate analyses for the outcome of depression can be seen in Table 3. The strongest predictor of depression was social support, such that high (-1.30, 95%CI, -1.44, -1.16) and medium levels (-0.73, 95%CI, -0.85, -0.62) of social support were protective against depression (relative to low levels) with a very large and large effect sizes, respectively. The only other large effect size was for psychological flexibility, which also served in a protective manner (-0.20, 95%CI, -0.22, -0.19). Moderate effect sizes of predictors associated with less depression symptoms were also observed for higher education levels (see Table 3). Moderate effect sizes of predictors associated with more depression were worse family functioning (0.29, 95%CI, 0.27, 0.32) and not being able to obtain all basic supplies (0.49, 95%CI, 0.27, 0.70).

**Table 3:** Predictors of depression.

	MSBS Score	
	Coefficient (95% CI)	Effect Size (95% CI)
**Socio Demographic Predictors**		
Sex		
Male	Ref	Ref
Female	0.08 (-0.03, 0.19)	0.04 (-0.01, 0.09)
Age	-0.03 (-0.03, -0.02)	-0.17 (-0.20, -0.14)
Employment		
Working (full time)	Ref	Ref
Working (part time)	-0.05 (-0.17, 0.08)	-0.02 (-0.08, 0.04)
Unemployed	0.28 (0.14, 0.40)	0.12 (0.06, 0.18)
On parental leave	-0.04 (-0.34, 0.26)	-0.02 (-0.16, 0.12)
Retired	0.24 (-0.02, 0.51)	0.11 (-0.01, 0.23)
Education		
Primary School	Ref	Ref
High School	-0.20 (-0.70, 0.30)	-0.09 (-0.32, 0.14)
Some College/University	-0.15 (-0.65, 0.35)	-0.09 (-0.32, 0.14)
Graduated from College/University	-0.44 (-0.94, 0.05)	-0.20 (-0.43, 0.02)
Master/Postgraduate	-0.62 (-1.11, -0.12)	-0.28 (-0.51, -0.05)
Doctoral level	-0.67 (-1.18, -0.16)	-0.31 (-0.54, -0.07)
Other	-0.43 (-0.99, 0.12)	-0.20 (-0.45, 0.06)
Marital status		
Single	Ref	Ref
In a relationship/engaged	-0.13 (-0.27, 0.00)	-0.06 (-0.13, 0.00)
Married	-0.16 (-0.34, 0.01)	-0.08 (-0.16, 0.01)
Divorced	0.04 (-0.20, 0.28)	0.02 (-0.09, 0.13)
Widower	-0.03 (-0.48, 0.41)	-0.02 (-0.23, 0.19)
Other	-0.06 (-0.45, 0.33)	-0.03 (-0.21, 0.16)
Living situation		
Live alone	Ref	Ref
Live with parents	-0.00 (-0.17, 0.16)	-0.00 (-0.08, 0.08)
Live with one of parents	0.09 (-0.14, 0.32)	0.04 (-0.06, 0.15)
Live with my own family	-0.16 (-0.33, 0.01)	-0.07 (-0.15, 0.00)
Live with friends/roommates	-0.24 (-0.47, -0.02)	-0.11 (-0.22, -0.01)
Having Children		
Yes	Ref	Ref
No	0.02 (-0.12, 0.16)	0.00 (-0.05, 0.07)
**Lockdown Predictors**		
Weeks in quarantine/self-isolation	-0.00 (-0.02, 0.02)	-0.00 (-0.03, 0.02)
Leave for work		
No	Ref	Ref
Once only	-0.12 (-0.31, 0.08)	-0.05 (-0.14, 0.04)
A couple of times	-0.19 (-0.32, -0.06)	-0.09 (-0.15, -0.03)
More than 3 times per week	-0.32 (-0.46, -0.18)	-0.15 (-0.21, -0.08)
Changes in finance		
Have gotten better	Ref	Ref
Stayed about the same	0.12 (-0.06, 0.30)	0.05 (-0.03, 0.13)
Have gotten worse	0.31 (0.12, 0.51)	0.14 (0.05, 0.23)
Obtain all basic supplies		
Yes	Ref	Ref
Νο	0.47 (0.26, 0.68)	0.21 (0.12, 0.30)
Inner living space (m2)	-0.00 (-0.00, 0.00)	-0.04 (-0.06, -0.01)
Outer space (m2)	0.00 (-0.00, 0.00)	0.00 (-0.02, 0.02)
**Social Predictors**		
Family Functioning Score	0.28 (0.26, 0.30)	0.23 (0.21, 0.25)
Perceived Social support		
Low	Ref	Ref
Moderate	-0.69 (-0.80, -0.58)	-0.31 (-0.35, -0.26)
High	-1.23 (-1.36, -1.10)	-0.54 (-0.60, -0.48)
**Psychological Predictors**		
Mindfulness	-0.13 (-0.14, -0.11)	-0.18 (-0.20, -0.16)
Psychological Flexibility	-0.19 (-0.21, -0.18)	-0.35 (-0.37, -0.33)

*Note*: Effect size interpretation: tiny (0–0.05); very small (0.05–0.10); small (0.10–0.20); medium (0.20–0.30); large (0.30–0.40); very large (>.40); Color code: light green–dark green (tiny–very large positive effect); light red–dark red (tiny–very large negative effect)

The amount of depression symptoms reported on average within countries was similar for most countries with the exception of one country with lower reported levels than average with a large effect size (Austria (-0.71, 95%CI, -0.95, -0.47)) and one with higher levels than average with a large effect size (USA (0.85, 95%CI, 0.58, 1.13)). See supporting information for information on each country (S2–S6 Tables).

#### Affect

Results of multivariate analyses for the outcome of affect can be seen in Table 4. With respect to positive affect, social support (high support vs low support (5.69, 95%CI, 5.23, 6.16) and psychological flexibility (0.77, 95%CI, 0.74, 0.81) were both predictors with very large effect sizes. Interestingly, those who left their house more than three times per week had higher levels of positive affect than those that did not leave their house for work (1.68, 95%CI, 1.18, 2.17), with a medium effect size. Higher education levels were associated with higher levels of positive affect with a medium to large effect size (see Table 4, PANAS-Positive).

**Table 4:** Predictors of affect.

	PANAS-Positive		PANAS-Negative	
	Coefficient (95% CI)	Effect Size (95% CI)	Coefficient (95% CI)	Effect Size (95% CI)
Socio Demographic Predictors				
Sex				
Male	Ref	Ref	Ref	Ref
Female	-1.19 (-1.57, -0.81)	-0.15 (-0.19, -0.10)	1.22 (0.85, 1.56)	0.16 (0.11, 0.21)
Age	0.09 (0.07, 0.11)	0.15 (0.12, 0.19)	-0.08 (-0.10, -0.06)	-0.15 (-0.18, -0.11)
Employment				
Working (full time)	Ref	Ref	Ref	Ref
Working (part time)	-0.41 (-0.86, 0.03)	-0.05 (-0.11, 0.00)	0.17 (-0.26, 0.61)	0.02 (-0.03, 0.08)
Unemployed	-1.13 (-1.59, -0.66)	-0.14 (-0.20, -0.08)	0.72 (0.27, 1.17)	0.10 (0.04, 0.15)
On parental leave	-0.24 (-1.31, 0.84)	-0.03 (-0.16, 0.11)	0.68 (-0.36, 1.72)	0.09 (-0.05, 0.23)
Retired	-1.12 (-2.06, -0.18)	-0.14 (-0.26, -0.02)	-0.28 (-1.19, 0.63)	-0.04 (-0.16, 0.08)
Education				
Primary School	Ref	Ref	Ref	Ref
High School	1.83 (0.06, 3.61)	0.23 (0.01, 0.46)	-2.16 (-3.87, -0.44)	-0.29 (-0.51, -0.06)
Some College/University	1.66 (-0.12, 3.43)	0.21 (-0.02, 0.43)	-1.75 (-3.47, -0.04)	-0.24 (-0.46, -0.01)
Graduated from College/University	1.98 (0.22, 3.73)	0.25 (0.03, 0.47)	-2.21 (-3.90, -0.51)	-0.30 (-0.52, -0.07)
Master/Postgraduate	2.41 (0.65, 4.44)	0.30 (0.08, 0.53)	-2.53 (-4.23, -0.83)	-0.35 (-0.57, -0.12)
Doctoral level	2.62 (0.79, 4.44)	0.33 (0.10, 0.56)	-2.09 (-3.85, -0.34)	-0.28 (-0.51, -0.04)
Other	1.87 (-0.11, 3.84)	0.24 (-0.01, 0.48)	-1.61 (-3.52, 0.29)	-0.21 (-0.47, 0.04)
Marital status				
Single	Ref	Ref	Ref	Ref
In a relationship/engaged	0.43 (-0.05, 0.90)	0.05 (-0.01, 0.12)	-0.12 (-0.58, 0.35)	-0.02 (-0.08, 0.05)
Married	0.32 (-0.31, 0.95)	0.04 (-0.04, 0.12)	-0.43 (-1.04, 0.17)	-0.06 (-0.14, 0.02)
Divorced	0.37 (-0.48, 1.22)	0.05 (-0.06, 0.16)	-0.89 (-1.71, -0.07)	-0.12 (-0.23, -0.01)
Widower	-0.22 (-1.79, 1.36)	-0.03 (-0.23, 0.17)	-0.65 (-2.18, 0.86)	-0.09 (-0.29, 0.11)
Other	-0.18 (-1.58, 1.22)	-0.02 (-0.20, 0.16)	-0.70 (-2.04, 0.65)	-0.09 (-0.27, 0.09)
Living situation				
Live alone	Ref	Ref	Ref	Ref
Live with parents	0.39 (-0.21, 1.00)	0.05 (-0.03, 0.13)	-0.01 (-0.56, 0.60)	-0.00 (-0.08, 0.08)
Live with one of parents	-0.31 (-1.13, 0.51)	-0.04 (-0.14, 0.06)	0.49 (-0.29, 1.28)	0.07 (-0.04, 0.17)
Live with my own family	0.69 (0.09, 1.29)	0.09 (0.01, 0.16)	-0.49 (-1.06, 0.09)	-0.06 (-0.14, 0.01)
Live with friends/roommates	1.13 (0.33, 1.93)	0.14 (0.04, 0.25)	0.15 (-0.62, 0.91)	0.02 (-0.08, 0.12)
Having children				
Yes	Ref	Ref	Ref	Ref
No	-0.61 (-1.11, -0.11)	-0.07 (-0.14, -0.01)	-1.23 (-1.71, -0.75)	-0.16 (-0.22, -0.10)
Lockdown predictors				
Weeks in quarantine /self-isolation	0.03 (-0.03, 0.09)	0.01 (-0.01, 0.04)	0.06 (-0.01, 0.11)	0.02 (0.00, 0.05)
Leave for work				
No	Ref	Ref	Ref	Ref
Once only	0.75 (0.06, 1.44)	0.10 (0.01, 0.19)	-0.48 (-1.13, 0.18)	-0.06 (-0.15, 0.02)
A couple of times	1.21 (0.74, 1.67)	0.16 (0.10, 0.22)	-0.73 (-1.17, -0.29)	-0.10 (-0.16, -0.04)
More than 3 times per week	1.68 (1.18, 2.17)	0.22 (0.15, 0.28)	-0.84 (-1.31, -0.37)	-0.11 (-0.18, -0.05)
Changes in finance				
Have gotten better	Ref	Ref	Ref	Ref
Stayed about the same	-0.56 (-1.20, 7.95)	-0.07 (-0.15, 0.01)	0.33 (-0.28, 0.941.23)	0.05 (-0.04, 0.13)
Have gotten worse	-1.03 (-1.71, -0.36)	-0.13 (-0.21, -0.04)	1.75 (1.10, 2.40)	0.24 (0.15, 0.33)
Obtain all basic supplies				
Yes	Ref	Ref	Ref	Ref
Νο	-1.34 (-2.09, -0.60)	-0.17 (-0.26, -0.08)	1.60 (0.89, 2.31)	0.21 (0.12, 0.30)
Inner living space (m2)	0.00 (0.00, 0.00)	0.07 (0.04, 0.09)	-0.00 (-0.00, 0.00)	-0.00 (-0.03, 0.02)
Outer space (m2)	0.00 (-0.00, 0.00)	0.03 (-0.02, 0.03)	-0.00 (-0.00, 0.00)	-0.02 (-0.05, 0.00)
Social Predictors				
Family Functioning Score	-0.81 (-0.90, -0.73)	-0.19 (-0.21, -0.17)	0.87 (0.79, 0.96)	0.21 (0.19, 0.23)
Perceived Social support				
Low	Ref	Ref	Ref	Ref
Moderate	3.11 (2.73, 3.49)	0.39 (0.34, 0.44)	-1.92 (-2.290, -1.54)	-0.22 (-0.27, -0.18)
High	5.69 (5.23, 6.16)	0.71 (0.65, 0.77)	-2.74(-3.20, -2.29)	-0.32 (-0.37, -0.27)
Psychological Predictors				
Mindfulness	0.59 (0.54, 0.64)	0.23 (0.21, 0.25)	-0.50 (-0.55, -0.45)	-0.21 (-0.23, -0.18)
Psychological Flexibility	0.77 (0.74, 0.81)	0.39 (0.37, 0.41)	-0.62 (-0.66, -0.58)	-0.33 (-0.35, -0.31)

*Note*: Effect size interpretation: tiny (0–0.05); very small (0.05–0.10); small (0.10–0.20); medium (0.20–0.30); large (0.30–0.40); very large (>.40); Color code: light green–dark green (tiny–very large positive effect); light red–dark red (tiny–very large negative effect).

The amount of positive affect reported on average within countries was similar for most countries with the exception of one country with lower reported levels than average with a large effect size (Finland (-2.96, 95%CI, -4.19, -1.73)) and one with higher reported levels than average with a large effect size (Portugal (2.96, 95%CI, 2.12, 3.80)). See supporting information for information on each country (S2–S6 Tables).

With respect to negative affect, social support (high support vs low support (-2.74, 95%CI, -3.2, -2.29) and psychological flexibility (-0.62, 95%CI, -0.66, -0.58) were again the strongest associated predictors, with large effects. Higher education levels were also associated with lower levels of negative affect, with a medium effect (see Table 4, PANAS-Negative). Higher levels of negative affect were noted, with medium effect sizes, for the predictors: worsening of finances (1.75, 95%CI, 1.10, 2.40) and not being able to obtain all basic supplies (1.6, 95%CI, 0.89, 2.31).

The amount of negative affect reported on average within countries was similar for most countries with the exception of few countries with lower reported negative affect levels than average with a very large effect sizes (Switzerland (-4.96, 95%CI, -5.91, -4.01), Germany (-4.70, 95%CI, -6.03, -3.37) & Austria (-6.49, 95%CI, -7.65, -5.33)) and one with a large effect size (Montenegro (-3.56, 95%CI, -5.39, -1.73). The average amount of negative affect was higher than average in two countries, with very large effects size (Turkey (5.75, 95%CI, 4.92, 6.59) & Finland (7.57, 95%CI, 5.80, 9.34)). See supporting information for information on each country (S2–S6 Tables).

#### Wellbeing

Results of multivariate analyses for the outcome of wellbeing can be seen in Table 5. Once again, social support (high support vs low support (13.20, 95%CI, 12.39, 14.01)) and psychological flexibility (1.42, 95%CI, 1.34, 1.49) were the predictors with the largest effect sizes (very large) on wellbeing. Higher education levels were associated with higher levels of wellbeing with a medium to large effect sizes (see Table 5). Medium negative effect sizes were noted for family functioning (-1.98, 95%CI, -2.12, -1.83) and inability to obtain all basic supplies (-3.27, 95%CI, -4.67, -1.87). Two medium positive effect sizes were observed: mindfulness (0.95, 95%CI, 0.86–1.04) and living with friends/roommates ((3.04, 95%CI, 1.59, 4.48), relative to living alone).

**Table 5:** Predictors of wellbeing.

	MHC-SF-Total Score	
	Coefficient (95% CI)	Effect Size (95% CI)
**Socio Demographic Predictors**		
Sex		
Male	Ref	Ref
Female	0.21 (-0.47, 0.91)	0.02 (-0.03, 0.07)
Age	0.13 (0.10, 0.17)	0.13 (0.10, 0.16)
Employment		
Working (full time)	Ref	Ref
Working (part time)	0.08 (-0.74, 0.91)	0.00 (-0.05, 0.07)
Unemployed	-1.95 (-2.80, -1.10)	-0.14 (-0.20, -0.08)
On parental leave	1.00 (-0.91, 2.92)	0.07 (-0.07, 0.21)
Retired	-0.43 (-2.13, 1.26)	-0.03 (-0.15, 0.09)
Education		
Primary School	Ref	Ref
High School	1.94 (-1.15, 5.03)	0.15 (-0.09, 0.38)
Some College/University	1.86 (-1.24, 4.97)	0.14 (-0.09, 0.38)
Graduated from College/University	2.36 (-0.69, 5.41)	0.18 (-0.05, 0.41)
Master/Postgraduate	3.34 (0.27, 6.40)	0.25 (0.02, 0.49)
Doctoral level	4.13 (0.95, 7.31)	0.31 (0.07, 0.56)
Other	2.65 (-0.83, 6.12)	0.20 (-0.06, 0.47)
Marital status		
Single	Ref	Ref
In a relationship/engaged	1.27 (0.39, 2.15)	0.10 (0.03, 0.16)
Married	1.99 (0.84, 3.14)	0.15 (0.06, 0.24)
Divorced	1.96 (0.41, 3.51)	0.15 (0.03, 0.26)
Widower	1.87 (-0.96, 4.71)	0.14 (-0.07, 0.36)
Other	-1.46 (-4.15, 1.22)	-0.11 (-0.31, 0.09)
Living Situation		
Live alone	Ref	Ref
Live with parents	2.22 (1.12, 3.32)	0.17 (0.08, 0.25)
Live with one of parents	-0.07 (-1.56, 1.40)	-0.00 (-0.12, 0.11)
Live with my own family	2.06 (0.96, 3.15)	0.15 (0.07, 0.24)
Live with friends/roommates	3.04 (1.59, 4.48)	0.23 (0.12, 0.34)
Having Children		
Yes	Ref	Ref
No	-1.11 (-2.02, -0.19)	-0.08 (-0.14, -0.01)
**Lockdown Predictors**		
Weeks in quarantine/self-isolation	-0.04 (-0.15, 0.07)	-0.00 (-0.04, 0.02)
Leave for work		
No	Ref	Ref
Once only	0.74 (-0.54, 2.01)	0.05 (-0.04, 0.15)
A couple of times	1.79 (0.94, 2.64)	0.13 (0.07, 0.20)
More than 3 times per week	2.56 (1.65, 3.47)	0.19 (0.12, 0.26)
Changes in finance		
Have gotten better	Ref	Ref
Stayed about the same	-0.96 (-2.10, 0.19)	-0.07 (-0.15, 0.01)
Have gotten worse	-2.17 (-3.39, -0.95)	-0.16 (-0.25, -0.07)
Obtain all basic supplies		
Yes	Ref	Ref
Νο	-3.27 (-4.67, -1.87)	-0.24 (-0.34, -0.14)
Inner living space (m2)	0.01 (0.00, 0.01)	0.09 (0.07, 0.12)
Outer space (m2)	0.00 (0.00, 0.00)	0.03 (0.00, 0.05)
**Social Predictors**		
Family Functioning Score	-1.98 (-2.12, -1.83)	-0.26 (-0.28, -0.24)
Perceived Social support		
Low	Ref	Ref
Moderate	7.78 (7.13, 8.43)	0.56 (0.52, 0.61)
High	13.20 (12.39, 14.01)	0.95 (0.90, 1.01)
**Psychological Predictors**		
Mindfulness	0.95 (0.86, 1.04)	0.22 (0.20, 0.24)
Psychological Flexibility	1.42 (1.34, 1.49)	0.42 (0.40, 0.44)

*Note*: Effect size interpretation: tiny (0–0.05); very small (0.05–0.10); small (0.10–0.20); medium (0.20–0.30); large (0.30–0.40); very large (>.40); Color code: light green–dark green (tiny–very large positive effect); light red–dark red (tiny–very large negative effect)

The level of wellbeing reported on average within countries was similar for most countries with the exception of three countries with higher levels with large effect sizes (Austria (4.95, 95%CI, 3.55, 6.34), Finland (5.24, 95%CI, 3.10, 7.38), & Portugal (4.59, 95%CI, 3.12, 6.05)) and two countries with lower levels of wellbeing than average with large (Italy (-4.36, 95%CI, -11.06, 2.35)) and very large effect sizes (Hong Kong (-6.84, 95%CI, -8.02, -5.66)). See supporting information for information on each country (S2–S6 Tables).

## Discussion

The COVID-19 is the largest pandemic in modern history. This study assessed nearly 10,000 participants across many countries to examine the impact of the pandemic and resultant governmental lockdown measures on mental health. During the height of the lockdown, the pandemic was experienced as at least moderately stressful for most people, and 11% reported the highest levels of stress. Symptoms of depression were also high, including 25% of the sample indicating that the things they did were not reinforcing, 33% reporting high levels of boredom, and nearly 50% indicating they wasted a lot of time. Consistent with symptoms of stress and depression, 10% of participants were psychologically languishing. These results suggest that there is a subgroup of people who are especially suffering and that in about 50% of the respondents’ levels of mental health was only moderate. Previous studies have found that along with low levels, even moderate levels of mental health (which consists of only moderate levels of emotional, psychological, and social well-being) are associated with increased subsequent disability, productivity loss, and healthcare use [35–37]. Not everyone was suffering, however, as evidenced by the nearly 40% of participants who reported levels of mental health consistent with flourishing. The present results, while serious, do not point to more severe reactions observed in previous samples of selective quarantined individuals or groups [2]. Perhaps the previously reported distress in these groups is prevented when an entire country or world is in lockdown so that the feeling emerges that “everyone is in it together”.

Importantly, a handful of predictors emerged that consistently predicted all outcomes: Social support, education level, finances, access to basic needs, and the ability to respond psychologically flexible. The consistency of results examining predictors is noteworthy, both in terms of the consistently strong predictors (e.g., social support, education, psychological flexibly, as well as loss of income and lack of access to necessities) and in terms of the other predictors that were either not predictive or only weakly so. All predictors were chosen based on theoretical ties to the outcomes, previous findings, and studies on quarantines [2].

A novel finding was that people who left their house three or more times per week reported more positive affect than those that left their house less often. It is possible that these people experienced more variation, which contributed to positive affect. It is also possible they experienced a greater sense of normality. Future studies are encouraged to further investigate possible mechanisms through which this result unfolds.

Overall, these patterns did not differ substantially between countries. Although some differences did emerge, they were mostly inconsistent across outcomes. Three countries fared worse on two outcomes each: Hong Kong (stress & wellbeing); Turkey (stress & negative affect); and Finland (lower positive affect and higher negative affect)–though participants in Finland also reported higher levels of wellbeing than average. Two countries had more favorable outcomes than the average levels across all countries: Portugal (lower stress and higher wellbeing) and Austria (lower depression and higher wellbeing). The differences observed are likely due to a combination of chance, sampling, nation specific responses to the COVID-19 pandemic, cultural differences, and other factors playing out in the countries (e.g., political unrest [38]). If replicated, future studies are encouraged to examine possible mechanisms of these outcomes.

This study provides valuable insights on several levels. First, it documents the mental health outcomes across a broad sample during the COVID-19 global pandemic. Second, it informs about the conditions and resilience factors (social support, education, and psychological flexibility) and risk factors (loss of income and inability to get basic supplies) that affect mental health outcomes. Third, these factors can be used in future public health responses are being made, including those that require large scale lockdowns or quarantines. That is, public health officials should direct resources to identifying and supporting people with poor social support, income loss, and potentially lower levels of education and provide a strategy to mitigate special risks in these subpopulations. The importance of social support needs to be made clear to the public and to the degree possible mechanisms that can contribute to social support should be supported. Further, psychological flexibility is a trainable set of skills that has repeatedly been shown to ameliorate suffering [22, 39]; and can be widely distributed with modern technological intervention tools such as digital, internet, or virtual means [40]. We do not claim, however, that psychological flexibility is the only factor that can be used for interventions. Instead, it is a recognized transdiagnostic factor assessed in this study and one that is feasible to be targeted and modified by interventions and prevention [41–43].

This study is limited by several important factors. First, the results are based on cross sectional analysis and correlations. As such, causation cannot be inferred and any delayed impact of the pandemic and lockdown on peoples’ mental health was not captured. Second, all results of this survey were obtained via self-report questionnaires, which can be subject to retrospective response bias. Third, although the sample was large and based on varied recruitment sources, it was not representative of the population and undersampled people who suffered most from the pandemic (i.e., front line health care professionals, people in intensive care, etc.) or people without internet access, etc. Finally, the country-specific incidence rates and lockdown measures differed across countries. These were not assessed, but future studies are encouraged to investigate how such factors impact mental health outcomes.

These limitations notwithstanding, based on nearly 10,000 international participants, this study found that approximately 10% of the population was languishing during or shortly after the lockdown period. These finding have implications for public health initiatives. First, officials are urged to attend to, find, and target people who have little social support and/ or whose finances have worsened as a result of the measures. Second, public health interventions are further urged to target psychological processes such as psychological flexibility in general to potentially help buffer other risk factors for mental health. Likewise, availability of social support and information about where to get support and remain connected are needed. These recommendations should become part of public health initiatives designed to promote mental health in general, and should equally be considered when lockdowns or physical distancing are prescribed during a pandemic.

## Supporting information

S1 Table List of all countries included in the data set. (PDF)

S2 Table Geodemographic predictors for Perceived Stress Scale. (PDF)

S3 Table Geodemographic predictors for MSBS–depression. (PDF)

S4 Table Geodemographic predictors for PANAS positive. (PDF)

S5 Table Geodemographic predictors for PANAS negative. (PDF)

S6 Table Geodemographic predictors for MHCSF—mental health continuum. (PDF)

S1 Appendix Participation flowchart. (PDF)
