# Supplementary material for: Impact of COVID-19 pandemic on mental health: An international study
Source: PLoS One. 2020 Dec 31;15(12):e0244809. doi: 10.1371/journal.pone.0244809 (PMC7774914; doi:10.1371/journal.pone.0244809)
Supplement: S1 Table — (PDF) [file pone.0244809.s001.pdf]

Supporting Information for: Impact of COVID-19 Pandemic on Mental Health: An International Study

S1 Table. List of all countries included in the data set.

Name of Country	n	Name of Country	n
Latvia	1285	Philippines	3
Italy	962	Peru	3
Cyprus	957	Iran	3
Turkey	702	China	3
Switzerland	550	United Arab Emirates	3
Hong Kong	516	Uruguay	3
Colombia	485	Russia	2
Ireland	414	Serbia	2
Austria	368	Malaysia	2
Romania	339	Angola	2
Portugal	334	South Africa	2
France	313	Norway	2
Spain	296	Pakistan	2
Germany	279	Jordan	2
Hungary	273	Azerbaijan	2
Greece	270	Ukraine	1
United States of America	268	Slovakia	1
Finland	157	South Korea	1
Montenegro	147	Ecuador	1
Poland	135	Thailand	1
United Kingdom	100	El Salvador	1
Slovenia	77	Lichtenstein	1
Canada	60	Lebanon	1
The Netherlands	30	Kuwait	1
Luxembourg	27	Croatia	1
Belgium	25	Namibia	1
Brazil	25	Guadeloupe	1
Vietnam	21	Saudi Arabia	1
Argentina	21	Paraguay	1
Australia	14	North Macedonia	1
Mexico	13	Iceland	1
Sweden	9	Belarus	1
Czech Republic	7	Uganda	1
India	7	Indonesia	1
New Zealand	5	Andorra	1
Chile	5	Panama	1
Denmark	3	Singapore	1
Israel	3	Taiwan	1
Cabo Verde	3		
