# Supplementary material for: Impact of COVID-19 pandemic on mental health: An international study
Source: PLoS One. 2020 Dec 31;15(12):e0244809. doi: 10.1371/journal.pone.0244809 (PMC7774914; doi:10.1371/journal.pone.0244809)
Supplement: S2 Table — (PDF) [file pone.0244809.s002.pdf]

S2 Table. Geodemographic predictors for Perceived Stress Scale.

		**Perceived Stress Scale**		
	**Mean (sd)**	**Difference between country mean and overall mean (95% CI)**	**Effect Size‡**	**Effect Size Interpretation**
**Variable**				
**Country**				
Cyprus	17.05 (7.53)	-0.03 (-0.49, 0.44)	-0.00	Tiny
Greece	16.68 (7.15)	-0.40 (-1.28, 0.47)	-0.06	Very small
Switzerland	16.30 (6.82)	-0.78 (-1.40, -0.16)	-0.11	Small
Germany	16.85 (6.64)	-0.23 (-1.10, 0.63)	-0.03	Tiny
Austria	15.84 (6.60)	-1.23 (-1.99, -0.48)	-0.17	Small
UK	17.47 (8.05)	0.39 (-1.05, 1.83)	0.05	Very small
Finland	16.49 (6.70)	-0.59 (-1.74, 0.57)	-0.08	Very small
Spain	15.95 (7.91)	-1.13 (-1.96, -0.29)	-0.16	Small
Ireland	15.94 (7.82)	-1.14 (-1.85, -0.43)	-0.16	Small
Italy	16.67 (6.53)	-0.41 (-0.88, 0.05)	-0.06	Very small
Latvia	17.68 (8.38)	0.60 (0.20, 1.00)	0.08	Very small
France	15.77 (7.68)	-1.32 (-2.13, -0.50)	-0.18	Small
Colombia	16.21 (8.52)	-0.86 (-1.52, -0.21)	-0.12	Small
Poland	18.47 (6.68)	1.38 (0.14, 2.63)	0.19	Small
Romania	17.29 (7.42)	0.21 (-0.58, 0.99)	0.03	Tiny
Hungary	16.88 (7.52)	-0.20 (-1.07, 0.68)	-0.03	Tiny
Portugal	14.59 (7.40)	-2.50 (-3.29, -1.71)	-0.35	Large
Turkey	19.55 (6.53)	2.47 (1.93, 3.02)	0.34	Large
USA	18.22 (7.96)	1.14 (0.26, 2.02)	0.16	Small
Hong Kong	19.93 (6.16)	2.85 (2.22, 3.49)	0.39	Large
Montenegro	13.78 (6.29)	-3.30 (-4.49, -2.11)	-0.46	Very Large
**‡ Cohen's d value for the standardize difference between the country mean and the overall mean**				

Note: For these analyses, only countries with n ≥ 100 participants were included
