# Supplementary material for: Impact of COVID-19 pandemic on mental health: An international study
Source: PLoS One. 2020 Dec 31;15(12):e0244809. doi: 10.1371/journal.pone.0244809 (PMC7774914; doi:10.1371/journal.pone.0244809)
Supplement: S3 Table — (PDF) [file pone.0244809.s003.pdf]

S3 Table. Geodemographic predictors for MSBS – Depression.

	**Mean (sd)**	**Difference between country mean and overall mean (95% CI)**	**Effect Size‡**	**Effect Size Interpretation**
**Variable**				
**Country**				
Cyprus	0.54 (2.28)	0.54 (0.40, 0.69)	0.23	Medium
Greece	0.33 (2.36)	0.34 (0.07, 0.62)	0.15	Small
Switzerland	-0.20 (2.27)	-0.19 (-0.38, 0.00)	-0.08	Very small
Germany	-0.16 (2.30)	-0.15 (-0.42, 0.12)	-0.07	Very small
Austria	-0.72 (2.18)	-0.71 (-0.95, -0.47)	-0.31	Large
UK	0.34 (2.66)	0.35 (-0.11, 0.80)	0.15	Small
Finland	-0.36 (2.18)	-0.35 (-0.71, 0.01)	-0.16	Small
Spain	0.24 (2.38)	0.25 (-0.02, 0.51)	0.11	Small
Ireland	-0.04 (2.32)	-0.03 (-0.25, 0.19)	-0.01	Tiny
Italy	-0.23 (2.27)	-0.22 (-0.36, -0.07)	-0.09	Very small
Latvia	-0.58 (2.44)	-0.57 (-0.70, -0.45)	-0.24	Small
France	-0.10 (2.32)	-0.09 (-0.35, 0.16)	-0.04	Tiny
Colombia	0.22 (2.40)	0.22 (0.02, 0.43)	0.10	Small
Poland	0.20 (2.14)	0.21 (-0.18, 0.60)	0.09	Very small
Romania	0.28 (2.15)	0.29 (0.05, 0.54)	0.13	Small
Hungary	-0.16 (2.38)	-0.15 (-0.42, 0.12)	-0.07	Very small
Portugal	-0.35 (2.06)	-0.35 (-0.59, -0.10)	-0.15	Small
Turkey	0.55 (2.44)	0.55 (0.38, 0.72)	0.24	Medium
USA	0.85 (2.51)	0.85 (0.58, 1.13)	0.38	Large
Hong Kong	0.20 (1.97)	0.21 (0.01, 0.41)	0.09	Small
Montenegro	-0.54 (2.12)	-0.54 (-0.91, -0.16)	-0.24	Medium
**‡ Cohen's d value for the standardize difference between the country mean and the overall mean**				

Note: For these analyses, only countries with n $\geq$ 100 participants were included
