# Supplementary material for: Impact of COVID-19 pandemic on mental health: An international study
Source: PLoS One. 2020 Dec 31;15(12):e0244809. doi: 10.1371/journal.pone.0244809 (PMC7774914; doi:10.1371/journal.pone.0244809)
Supplement: S4 Table — (PDF) [file pone.0244809.s004.pdf]

S4 Table. Geodemographic predictors for PANAS Positive.

	**Mean (sd)**	**Difference between country mean and overall mean (95% CI)**	**Effect Size‡**	**Effect Size Interpretation**
**Variable**				
**Country**				
Cyprus	30.87 (8.86)	1.82 (1.32, 2.31)	0.22	Medium
Greece	31.21 (8.15)	2.16 (1.23, 3.10)	0.28	Medium
Switzerland	29.35 (7.34)	0.30 (-0.35, 0.96)	0.04	Tiny
Germany	28.90 (7.43)	-0.15 (-1.07, 0.77)	-0.02	Tiny
Austria	30.44 (7.24)	1.40 (0.59, 2.20)	0.18	Tiny
UK	28.35 (9.40)	-0.70 (-2.24, 0.84)	-0.09	Very Small
Finland	26.09 (5.36)	-2.96 (-4.19, -1.73)	-0.39	Large
Spain	29.98 (8.28)	0.93 (0.04, 1.83)	0.12	Small
Ireland	28.35 (8.97)	-0.70 (-1.45, 0.06)	-0.09	Very Small
Italy	28.42 (7.18)	-0.63 (-1.13, -0.13)	-0.08	Very Small
Latvia	27.38 (7.66)	-1.66 (-2.09, -1.23)	-0.20	Medium
France	29.60 (7.18)	0.54 (-0.33, 1.41)	0.07	Very Small
Colombia	30.36 (8.82)	1.31 (0.61, 2.01)	0.17	Small
Poland	25.59 (7.97)	-3.46 (-4.78, -2.13)	-0.45	Very Large
Romania	29.42 (7.21)	0.38 (-0.46, 1.21)	0.05	Very Small
Hungary	30.89 (7.99)	1.84 (0.91, 2.77)	0.24	Medium
Portugal	32.01 (7.43)	2.96 (2.12, 3.80)	0.38	Large
Turkey	30. 47 (8.17)	1.42 (0.84, 2.00)	0.18	Small
USA	24.83 (8.48)	-4.22 (-5.16, -3.28)	-0.54	Very Large
Hong Kong	25.66 (7.22)	-3.38 (-4.06, -2.71)	-0.43	Very Large
Montenegro	30.77 (6.36)	1.72 (0.45, 2.99)	0.22	Medium
**‡ Cohen's d value for the standardize difference between the country mean and the overall mean**				

Note: For these analyses, only countries with n ≥ 100 participants were included
