# Supplementary material for: Impact of COVID-19 pandemic on mental health: An international study
Source: PLoS One. 2020 Dec 31;15(12):e0244809. doi: 10.1371/journal.pone.0244809 (PMC7774914; doi:10.1371/journal.pone.0244809)
Supplement: S5 Table — (PDF) [file pone.0244809.s005.pdf]

**S5 Table.** Geodemographic predictors for PANAS Negative.

| | | | | |
|---|---|---|---|---|
| | | | | |
| | | | | |
| | **Mean (sd)** | **Difference between country mean and overall mean (95% CI)** | **Effect Size‡** | **Effect Size Interpretation** |
| **Variable** | | | | |
| **Country** | | | | |
| Cyprus | 30.07 (11.64) | -0.32 (-1.04, 0.40) | -0.03 | Tiny |
| Greece | 30.35 (11.04) | -0.03 (-1.38, 1.32) | 0.00 | Tiny |
| Switzerland | 25.42 (8.91) | -4.96 (-5.91, -4.01) | -0.44 | Very Large |
| Germany | 25.68 (8.46) | -4.70 (-6.03, -3.37) | -0.42 | Very Large |
| Austria | 23.90 (8.39) | -6.49 (-7.65, -5.33) | -0.58 | Very Large |
| UK | 29.32 (10.56) | -1.06 (-3.29, 1.16) | -0.09 | Very Small |
| Finland | 37.95 (14.61) | 7.57 (5.80, 9.34) | 0.67 | Very Large |
| Spain | 31.53 (10.98) | 1.15 (-0.14, 2.44) | 0.10 | Small |
| Ireland | 28.05 (10.74) | -2.33 (-3.43, -1.24) | -0.21 | Medium |
| Italy | 31.33 (11.16) | 0.95 (0.23, 1.66) | 0.08 | Very Small |
| Latvia | 30.50 (12.20) | 0.12 (-0.50, 0.74) | 0.01 | Tiny |
| France | 30.87 (12.32) | 0.49 (-0.76, 1.75) | 0.04 | Tiny |
| Colombia | 32.73 (11.58) | 2.34 (1.33, 3.35) | 0.20 | Medium |
| Poland | 27.74 (10.92) | -2.64 (-4.56, -0.73) | -0.24 | Medium |
| Romania | 31.55 (12.11) | 1.16 (-0.05, 2.37) | 0.10 | Small |
| Hungary | 27.17 (10.95) | -3.22 (-4.56, -1.87) | -0.28 | Medium |
| Portugal | 27.98 (10.15) | -2.40 (-3.61, -1.18) | -0.21 | Medium |
| Turkey | 36.14 (12.55) | 5.75 (4.92, 6.59) | 0.49 | Very Large |
| USA | 31.97 (11.90) | 1.59 (0.23, 2.94) | 0.14 | Small |
| Hong Kong | 33.54 (12.38) | 3.15 (2.18, 4.13) | 0.27 | Medium |
| Montenegro | 26.82 (8.48) | -3.56 (-5.39, -1.73) | -0.32 | Large |
| | | | | |
| **‡ Cohen's d value for the standardize difference between the country mean and the overall mean** | | | | |

Note: For these analyses, only countries with n ≥ 100 participants were included
