# Supplementary material for: Impact of COVID-19 pandemic on mental health: An international study
Source: PLoS One. 2020 Dec 31;15(12):e0244809. doi: 10.1371/journal.pone.0244809 (PMC7774914; doi:10.1371/journal.pone.0244809)
Supplement: S6 Table — (PDF) [file pone.0244809.s006.pdf]

S6 Table. Geodemographic predictors for MHCSF - Mental Health Continuum.

	**Mean (sd)**	**Difference between country mean and overall mean (95% CI)**	**Effect Size‡**	**Effect Size Interpretation**
**Variable**				
**Country**				
Cyprus	41.63 (14.36)	0.59 (-0.28, 1.46)	0.04	Tiny
Greece	41.41 (13.94)	0.37 (-1.26, 2.00)	0.03	Tiny
Switzerland	43.41 (13.22)	2.37 (1.22, 3.51)	0.17	Small
Germany	43.72 (12.51)	2.68 (1.07, 4.28)	0.20	Medium
Austria	45.98 (12.33)	4.95 (3.55, 6.34)	0.37	Large
UK	39.67 (14.31)	-1.37 (-4.10, 1.37)	-0.10	Small
Finland	46.28 (12.65)	5.24 (3.10, 7.38)	0.39	Large
Spain	41.92 (13.26)	0.89 (-0.68, 2.45)	0.07	Very small
Ireland	42.49 (15.33)	1.45 (0.13, 2.77)	0.11	Small
Italy	36.68 (15.69)	-4.36 (-11.06, 2.35)	-0.33	Large
Latvia	40.40 (13.49)	-0.64 (-1.39, 0.11)	-0.05	Very small
France	39.36 (12.84)	-1.68 (-3.20, -0.16)	-0.12	Small
Colombia	43.10 (14.83)	2.06 (0.84, 3.27)	0.15	Small
Poland	34.28 (14.24)	-6.76 (-9.07, -4.45)	-0.50	Very large
Romania	38.27 (14.07)	-2.77 (-4.23, -1.31)	-0.20	Medium
Hungary	37.26 (13.89)	-3.78 (-5.41, -2.16)	-0.28	Medium
Portugal	45.62 (12.38)	4.59 (3.12, 6.05)	0.34	Large
Turkey	39.13 (13.88)	-1.91 (-2.92, -0.89)	-0.14	Small
USA	41.25 (14.93)	0.21 (-1.42, 1.85)	0.02	Tiny
Hong Kong	34.19 (12.52)	-6.84 (-8.02, -5.66)	-0.50	Very Large
Montenegro	44.48 (12.57)	3.45 (1.23, 5.66)	0.26	Medium
**‡ Cohen's d value for the standardize difference between the country mean and the overall mean**				

Note: For these analyses, only countries with n $\geq$ 100 participants were included
