# Supplementary figures and images for: Impact of COVID-19 pandemic on mental health: An international study
Source: PLoS One. 2020 Dec 31;15(12):e0244809. doi: 10.1371/journal.pone.0244809 (PMC7774914; doi:10.1371/journal.pone.0244809)

**S1 Appendix.** Participation Flowchart.

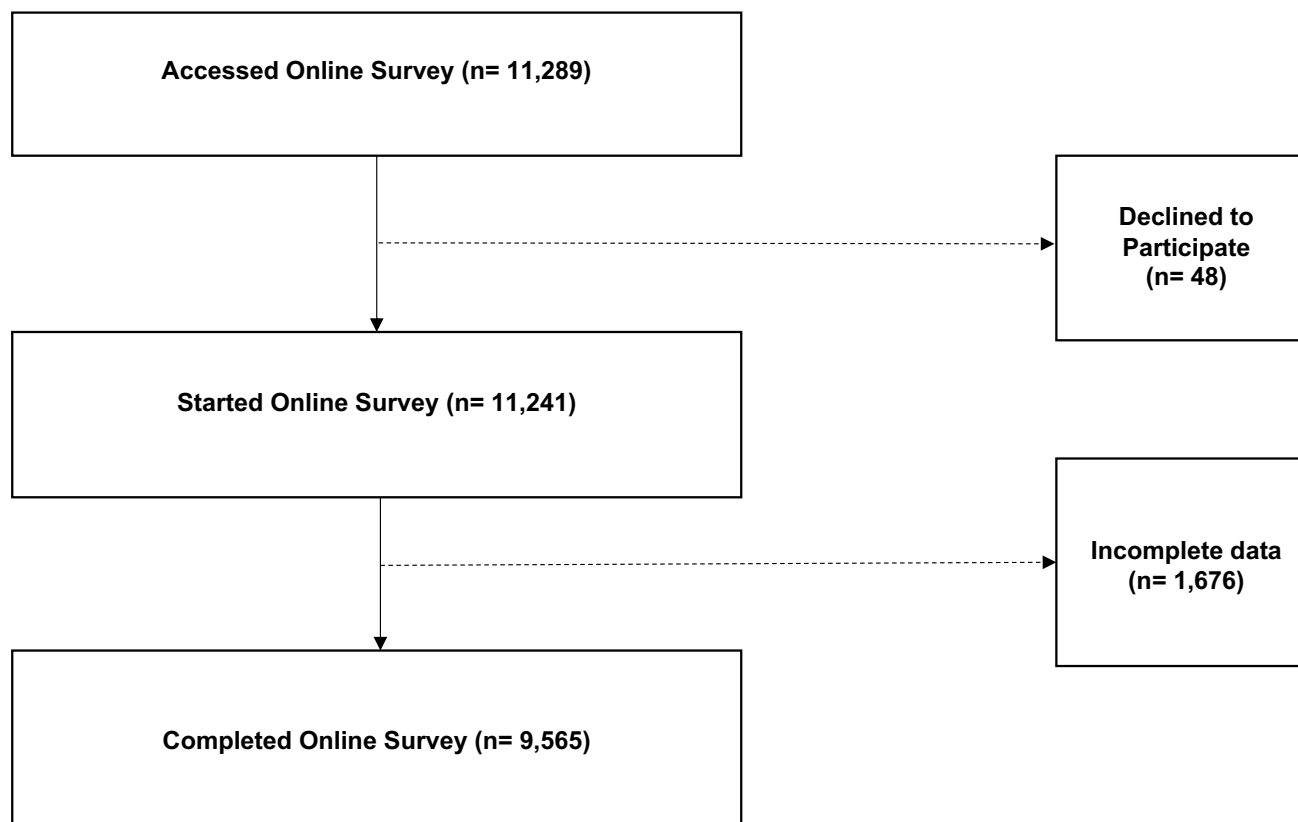

Supplement: S1 Appendix — (PDF) [file pone.0244809.s007.pdf]
